# gtAI: an improved species-specific tRNA adaptation index using the genetic algorithm

**DOI:** 10.3389/fmolb.2023.1218518

**Published:** 2023-07-04

**Authors:** Ali Mostafa Anwar, Saif M. Khodary, Eman Ali Ahmed, Aya Osama, Shahd Ezzeldin, Anthony Tanios, Sebaey Mahgoub, Sameh Magdeldin

**Affiliations:** ^1^ Proteomics and Metabolomics Research Program, Basic Research Department, Children’s Cancer Hospital 57357 (CCHE-57357), Cairo, Egypt; ^2^ Department of Genetics, Faculty of Agriculture, Cairo University, Giza, Egypt; ^3^ Department of Pharmacology, Faculty of Veterinary Medicine, Suez Canal University, Ismailia, Egypt; ^4^ Department of Physiology, Faculty of Veterinary Medicine, Suez Canal University, Ismailia, Egypt

**Keywords:** codon usage, tRNA adaptation index, molecular evolution, translational selection, codon usage analysis

## Abstract

The tRNA adaptation index (tAI) is a translation efficiency metric that considers weighted values (*S*_*ij*_ values) for codon–tRNA wobble interaction efficiencies. The initial implementation of the tAI had significant flaws. For instance, generated *S*_*ij*_ weights were optimized based on gene expression in *Saccharomyces cerevisiae*, which is expected to vary among different species. Consequently, a species-specific approach (stAI) was developed to overcome those limitations. However, the stAI method employed a hill climbing algorithm to optimize the *S*_*ij*_ weights, which is not ideal for obtaining the best set of *S*_*ij*_ weights because it could struggle to find the global maximum given a complex search space, even after using different starting positions. In addition, it did not perform well in computing the tAI of fungal genomes in comparison with the original implementation. We developed a novel approach named genetic tAI (gtAI) implemented as a Python package (https://github.com/AliYoussef96/gtAI), which employs a genetic algorithm to obtain the best set of *S*_*ij*_ weights and follows a new codon usage-based workflow that better computes the tAI of genomes from the three domains of life. The gtAI has significantly improved the correlation with the codon adaptation index (CAI) and the prediction of protein abundance (empirical data) compared to the stAI.

## 1 Introduction

Grantham et al. (1980) highlighted the unequal usage of synonymous codons among different genes and genomes in a phenomenon currently known as codon usage bias (CUB). Thenceforward, scientists were investigating the effect of synonymous mutations on the efficiency/accuracy of protein translation and several biological processes ranging from RNA processing to protein folding and their potential consequences on the overall performance and evolution of living organisms (Chamary et al., 2006; Plotkin and Kudla, 2011). Codon usage is positively associated with the analogous tRNA in a species—the tRNA pool determines the available amino acid used during the protein extension process. Therefore, protein expression and translation efficiency are highly associated with CUB (Karlin et al., 2001; Gustafsson et al., 2004). Accordingly, codons with high occurrence in a gene (putative optimal codons) improve the protein translation rate, and rare codons will cause a reduction in the translation and might cause translation errors (Ikemura, 1981).

In biotechnology studies, heterologous expression was applied to assemble vaccines and pharmaceuticals (Han et al., 2010; Liu et al., 2018). Codon optimization was proposed to increase heterologous gene expression (Quax et al., 2015; Brandis and Hughes, 2016; Fu et al., 2020). Many studies reported the success of the codon optimization approach to upregulate gene expression up to 1,000-fold (Quax et al., 2015). Several software tools are used for codon optimization and are patented to serve commercial purposes such as GenSmart Design (https://www.genscript.com/gene-and-plasmid-construct-design.html) and GENEWIZ (https://www.genewiz.com/en-GB/Public/Services/Gene-Synthesis/codon-optimization). A number of codon optimization algorithms are not open-source (Satya et al., 2003; Huang et al., 2021) or should be requested from the authors (Fuglsang, 2003). Regardless of their availability, many of those protein expression optimization software tools are based on the tRNA adaptation index (tAI) (Gould et al., 2014; Watts et al., 2021; Raguin et al., 2023) and codon adaptation index (CAI) (Fu et al., 2020). Many indices were developed to measure the degree of preference for the unbalanced use of codons. Some are codon-specific such as relative synonymous codon usage (RSCU), and others are gene-specific such as the effective number of codons (ENc) (Wright, 1990; Sun et al., 2013) and CAI (Sharp and Li, 1987). A relatively new index named tAI was introduced by dos Reis et al. (2004) to become a formal measure for CUB associated with translational selection. The tRNA presents a complementary anticodon for an amino acid to be incorporated into the growing polypeptide chain during the translation process. The codon–anticodon interactions at the first two codon positions are governed solely by canonical (Watson–Crick) base pairing rules, unlike the third codon position at which non-canonical (wobble) base pairing also occurs (Crick, 1966). The tAI considers weights for canonical and wobble interaction efficiencies between codons and tRNA molecules. To compute the tAI, first, the absolute adaptiveness value (*W*_*i*_) for codon *i* is calculated by the following equation:

$$W_{i}=\sum_{j=1}^{n_{j}}\left(1-S_{ij}\right){tGNC}_{ij},$$ (1)

where *n*_*j*_ is the number of tRNA isoacceptors that can recognize the *i*th codon, *S*_*ij*_ is the codon–anticodon coupling efficiency having values ranging from 0 (perfect interaction) to 1 (weak interaction) (dos Reis et al., 2004), and *tGNC*_*ij*_ is the gene copy number of the *j*th anticodon that can recognize the *i*th codon.

Then, each *W*_*i*_ is normalized to the maximum *W*_*i*_ value to obtain the relative adaptiveness value (*w*_*i*_). Finally, the tAI of a gene can be defined as the geometric mean of the *w*_*i*_ values of its codons (dos Reis et al., 2004):

$$tAI_{g}=\exp\left(\frac{1}{O_{tot}}\underset{i\in I}{\sum }\log\;w_{i}\right),$$ (2)

where *O*_*tot*_ is the frequency of the total codons.

The *S*_*ij*_ weights inferred by the original tAI (otAI) implementation were based on optimizing the correlation between tAI (Eq. 1) and gene expression levels in *Saccharomyces cerevisiae* using the Nelder–Mead method under the assumption that highly expressed genes contain codons with higher adaptation to the tRNA pool (driven by the force of translational selection). In a study by Dana and Tuller (2014), two problems are associated with the original tAI implementation. First, it depends on gene expression information, often unavailable for many organisms (especially novel ones). Second, generated weights were specific for *Saccharomyces cerevisiae*. They suggested the possibility that wobble interaction efficiencies shall differ significantly among genomes from different domains. So, it would not be plausible to use the weights specifically for *Saccharomyces cerevisiae* to compute the tAI of other organisms. Consequently, they developed the species-specific tAI (stAI) (Sabi and Tuller, 2014) to solve these problems.

The inferred stAI weights are based on optimizing the correlation between the tAI (Eq. 1) and a CUB index, namely, directional codon bias score (a modified version of relative CUB (Oymondal et al., 2009)) using the hill climbing algorithm under the assumption that highly expressed genes have higher adaptation to the tRNA pool and higher CUB (Sabi and Tuller, 2014). This eliminates the need for additional gene expression data and generates weights specific to the tested organism, indicating the value of stAI in tAI computation, especially for non-fungal species. However, two limitations in the stAI are as follows: 1) using the hill climbing optimization method by which only local maxima can be reached and often gets stuck in ridges and plateau scenarios (Thengade and Dondal, 2012); hence, the best set of *S*_*ij*_ weights may not be obtained even after using different starting positions (random restart) (Russell and Norvig, 2010; Yang, 2014); 2) the outperformance of the original tAI over the stAI in predicting the protein abundance (PA) of fungal organisms (Sabi and Tuller, 2014).

Here, we introduce a novel approach for tAI computation, namely, genetic tAI (gtAI), to solve the problems associated with the stAI, which affect its performance. The gtAI uses a genetic algorithm to reach the global maximum (best set of *S*_*ij*_ weights), solving the issue of obtaining a meaningful set of *S*_*ij*_ weights for each organism. It also utilizes robust CUB indices (ENc and RSCU) different from the directional codon bias score (DCBS) employed by the stAI.

## 2 Materials and methods

### 2.1 Establishing a reference set of genes using the effective number of codons

A reference set of genes is defined as a set of genes with the highest expression levels in a genome, such as ribosomal genes and translation elongation factors (Duret, 2000; Ghaemmaghami et al., 2003; Goetz and Fuglsang, 2005). The ENc is a widely used measure of CUB at the gene level, and in theory, it negatively correlates with gene expression (Sun et al., 2013). Given the assumption that highly expressed genes are highly biased (Sabi and Tuller, 2014), a reference set is obtained by selecting genes with the lowest ENc values (highest expression) in the tested genome. The ENc is calculated using the equations of the improved ENc implementation by Sun et al. (2013):

$${EN}_{c}=N_{s}+\frac{K_{2}\sum_{j}^{k_{2}}nj}{\sum_{j=1}^{k_{2}}\left(njF_{CF.j}\right)}+\frac{K_{3}\sum_{j}^{k_{3}}nj}{\sum_{j=1}^{k_{3}}\left(njF_{CF.j}\right)}+\frac{K_{4}\sum_{j}^{k_{4}}nj}{\sum_{j=1}^{k_{4}}\left(njF_{CF.j}\right)},$$ (3)

where *N*_*S*_ is the number of codon families with a single codon. *K*_*i*_ is the number of *i*-fold codon families. In addition, *F*_*CF. j*_ is *F*_*CF*_ for family *j* obtained from the following equation:

$$F_{CF}=\sum_{i=1}^{m}{\left(\frac{n_{i}+1}{n+m}\right)}^{2},$$ (4)

where *n*_*i*_ is the count of codon *i* in the codon family of m synonymous codons.

### 2.2 Calculating the relative synonymous codon usage for the reference set

The RSCU is a codon-specific CUB measurement defined as the ratio of the observed to the expected frequency of codons, under the null hypothesis that all synonymous codons for a particular amino acid are used equally (Sharp and Li, 1986). It gives an accurate value for each amino acid codon ranging from 0 to the number of synonymous codons for that amino acid. The RSCU values for the reference set are calculated using the following equation:

$$RSCU=\frac{O_{ac}}{\frac{1}{k_{a}}\underset{c\in C_{a}}{\sum }O_{ac}},$$ (5)

where *O*_*ac*_ is the count of codon *c* for the amino acid *a* and *k*_*a*_ is the number of synonymous codons in the amino acid *a* family.

### 2.3 *S*_*ij*_ weight inference by the genetic algorithm

Since highly expressed genes are influenced by translational selection to include more codons with higher adaptation to the intracellular tRNA pool (i.e., optimal codons) (Sabi and Tuller, 2014), we expect to find a correlation between RSCU (Eq. 5) and absolute adaptiveness (*W*_*i*_) values (Eq. 1). Therefore, we inferred unique *S*_*ij*_ weights for each organism by optimizing the non-parametric (Spearman’s rank) correlation between RSCU (of the reference set) and *W*_*i*_ values using a genetic algorithm (https://pypi.org/project/gaft/). It should be noted that the correlation between RSCU and *W*_*i*_ is at the level of codons.

The genetic algorithm is a metaheuristic search approach inspired by the Darwinian principle of survival of the fittest. It will search for the best *S*_*ij*_ weights that maximize the correlation between RSCU and *W*_*i*_ while operating in Algorithm 1

Algorithm 1 The genetic algorithm operates to optimize the Sij weights used to calculate the tAI values.

**Input:** Genome coding sequences
Initialize S, vector of the initial population as chromosomes (*S*_*ij*_ sets) with random *S*_*ij*_ values (genes)
Generation time = n;
F**or** s in S **do**
Evaluate fitness function(s);
n + = 1
IntialLabel;
**Test:**
Selection(s) where Sij sets that exhibit higher correlation between RSCU and Wi;
**Do:**
Crossover(s);
Mutation(s);
Evaluate fitness function(s);
**If** n = Generation time, **then**
Output = Best fitness(s);
**Else**
Go to IntialLabel
**Output:** the best set of *S*_*ij*_ weights + tAI values

Then, the best set of *S*_*ij*_ weights will be used to compute the tAI values using Eqs. 1, 2 (Sabi and Tuller, 2014).

### 2.4 Genomic data collection

The coding sequences of 12 organisms (*Ferroglobus placidus*, *Halomicrobium mukohataei*, *Methanocaldococcus jannaschii*, *Escherichia coli*, *Neisseria meningitides*, *Vibrio cholera*, *Caenorhabditis elegans*, *Drosophila melanogaster*, *Aspergillus fumigatus*, *Aspergillus nidulans*, *Saccharomyces cerevisiae*, and *Schizosaccharomyces pombe*) used in this study as representatives of different domains were retrieved from NCBI (https://www.ncbi.nlm.nih.gov/genome/) in the FASTA format. Their tRNA gene copy numbers were obtained from GtRNAdb (Chan and Lowe, 2009). All information about the used organisms can be found in Supplementary Table S1.

### 2.5 CAI and the original tAI indices’ calculation

The CAI was calculated using a Python package (Lee, 2018). In addition, the original tAI was calculated using a Python code developed by the authors (the same used to calculate the tAI in the gtAI package) using the *S*_*ij*_ weights found in the original study (Sabi and Tuller, 2014).

### 2.6 Protein abundance data collection

To test to what extent gtAI correlates with empirical data such as PA compared to the otAI and stAI, the PA data of *E. coli*, *C. elegans*, *D. melanogaster*, *S. cerevisiae*, and *S. pombe* were retrieved from PaxDB. The integrated PaxDB version (highest coverage) was used for all the organisms (version 4.1) (Wang et al., 2015). These organisms were chosen due to the availability of their PA data.

### 2.7 The impact of generation time and population size parameter choice on gtAI result reproducibility

First, we investigated the effect of the population size parameter on the gtAI result. Three random organisms were selected from the 12 used in this study (*S. cerevisiae*, *E. coli*, and *H. mukohataei*). For each organism, the non-parametric (Spearman) correlation between RSCU (of the reference set) and *W*_*i*_ values were optimized by the genetic algorithm used in gtAI calculation. We chose a constant generation time equal to 100 and different population sizes (10, 20, 30, n + 10 … , 100). Hence, each organism was optimized 10 times, each with a different population size to compare the best solution between each population size (inter-variability). This experiment was performed for each organism five times to reach the best solution within the same population size and for the same organism between different experiments (intra-variability). Then, we tested the best solution for selecting the suitable generation time by applying 1,000 generations on the same three organisms, with a constant population size equal to 60. Finally, we plotted the solutions from generation 1 to 1,000.

## 3 Results

### 3.1 CAI correlations with gtAI, stAI, and otAI

The Williams’ test was used to compare the rho values at an alpha score of 0.01 (two-sided test). The gtAI values for *H. mukohataei*, *M. jannaschii*, *E. coli*, *N. meningitidis*, *D. melanogaster*, *A. nidulans*, *S. pombe*, *A. fumigatus*, and *S. cerevisiae* (9 out of 12) revealed a statistically significant (Williams’ test *p* value <0.01) higher correlation with CAI than stAI (Table 1). Moreover, the gtAI in all the fungal organisms showed a higher considerable correlation (*A. nidulans*, *S. pombe*, *A. fumigatus*, and *S. cerevisiae*) with the CAI than the original tAI (Supplementary Tables S1–S4).

**TABLE 1:** Spearman’s rank correlation analysis between CAI and the three tAI measurements (original tAI, stAI, and gtAI) for the 12 model organisms and their average GC content and ENc values.

		gtAI-CAI (rho)	stAI-CAI (rho)	tAI-CAI (rho)	GC content (%)	ENc
Archaea						
	*Ferroglobus placidus*	0.46*	0.48*	-	44.1	44.81
	*Halomicrobium mukohataei*	0.61*	0.41*	-	65.5	34.26
	*Methanocaldococcus jannaschii*	0.23*	0.14*	-	31.4	37.92
Bacteria						
	*Escherichia coli*	0.83*	0.82*	-	50.8	39.09
	*Neisseria meningitidis*	0.9*	0.67*	-	51.8	36.89
	*Vibrio cholera*	0.78*	0.8*	-	48.1	41.76
Eukarya (non-fungal)						
	*Caenorhabditis elegans*	0.8*	0.82*	-	35.4	42.02
	*Drosophila melanogaster*	0.89*	0.74*	-	42.0	38.69
Eukarya (fungal)						
	*Aspergillus fumigatus*	0.91*	0.78*	0.82*	49.5	40.64
	*Aspergillus nidulans*	0.94*	0.29*	0.91*	50.1	41.57
	*Saccharomyces cerevisiae*	0.94*	0.56*	0.87*	38.2	36.42
	*Schizosaccharomyces pombe*	0.88*	0.56*	0.84*	36	40.32

* represents *p* value <0.001.

### 3.2 Repeated random sampling and correlations with the CAI

First, we calculated the gtAI, stAI, and CAI values for the genes of all tested organisms. Then, for each replicate (1,000 replicates with replacement), we sampled a 25% random sample size from the values of these measures for the Spearman’s rank correlation analyses. This is to make sure that the reference set of genes present among other genes is not causing inflated gtAI-CAI correlations. The rep_sample_n R function from the infer package was used in the random sampling (https://www.rdocumentation.org/packages/infer/versions/1.0.4/topics/rep_sample_n). It does not specify a particular distribution type to be used but rather allows for repeated sampling of data from a specified data frame. The script of this random sampling method could be found here (https://github.com/AliYoussef96/gtAI/blob/master/random%20sampling.r).

**FIGURE 1:**
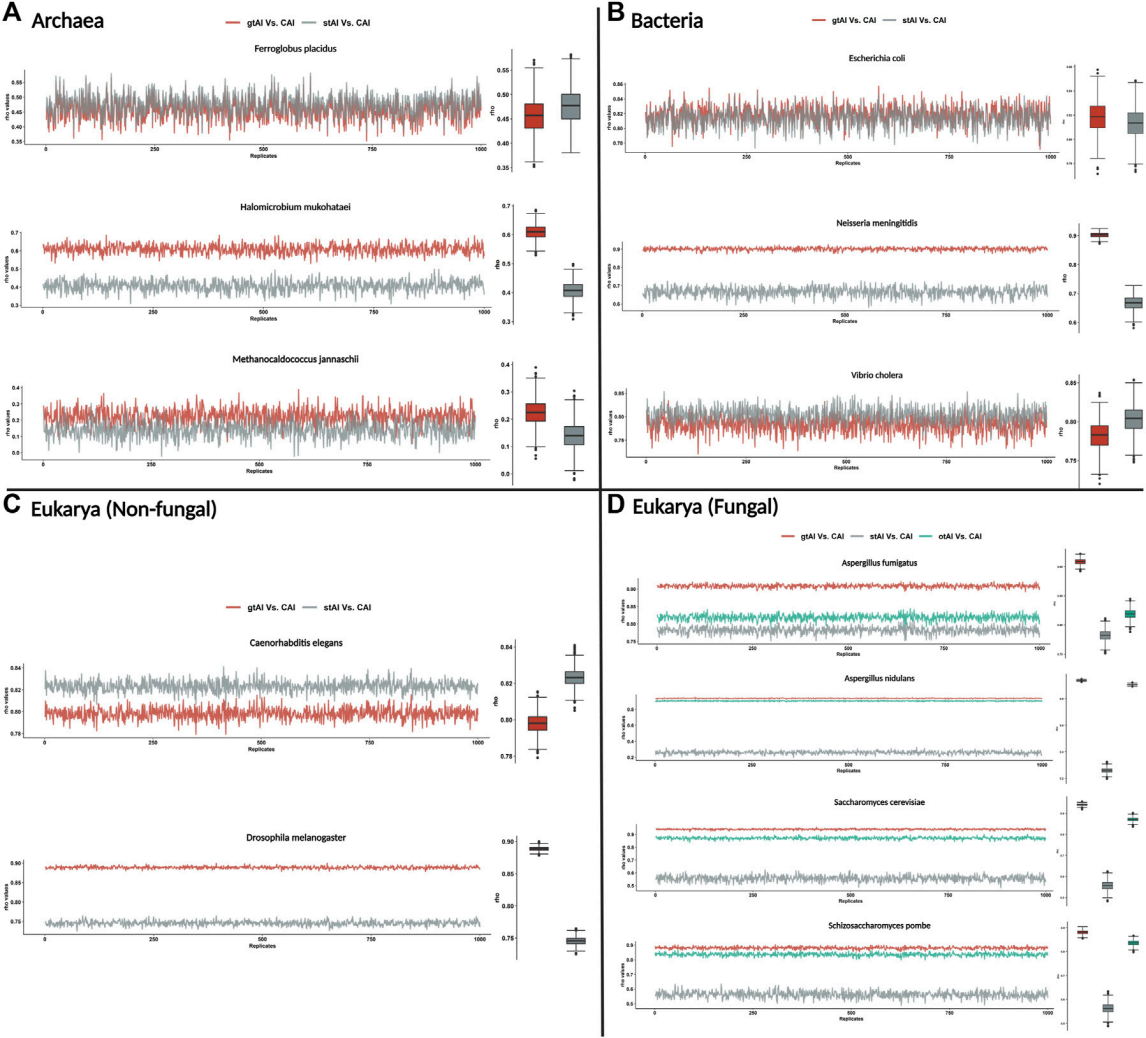
Repeated Spearman’s rank correlation analysis between gtAI-CAI, stAI-CAI, and otAI-CAI for randomly sampled values. The gtAI, stAI, and CAI values for each organism (the 12 used in the study) were randomly sampled with size 25%, and 1,000 replicates with replacement have been taken. Spearman’s rank correlation analysis between gtAI-CAI, stAI-CAI, and otAI-CAI for each replicate was applied. The line plot shows the rho values for each replicate, and the box plots show the distributions. **(A)** Three archaea organisms being tested in this study, **(B)** the three bacterial organisms, **(C)** two eukaryotic (non-fungal) organisms, and **(D)** four eukarya (fungal) organisms. The color code is red for gtAI-CAI correlation results, gray for stAI-CAI correlation results, and green for the original tAI (otAI)-CAI correlation results.

The result showed that the gtAI exhibited stronger correlations for the same nine organisms compared to the stAI. Furthermore, a stronger correlation with the CAI in the four fungal organisms compared to the otAI is shown (Figure 1).

### 3.3 SCUO correlations with gtAI, stAI, and otAI

The SCUO is a codon usage index that does not involve the use of a reference set in its calculation (Wan et al., 2004). The gtAI, stAI, otAI, and SCUO values were calculated for all 12 organisms. The gtAI has outperformed both stAI and otAI by exhibiting a stronger statistically significant correlation with SCUO in eight organisms consistent with CAI association results except in *E. coli*, and the gtAI-SCUO and stAI-SCUO correlations are 0.26 and 0.27, respectively (Table 2). A two-sided Williams’ test was used to compare the rho values at an alpha score of 0.01.

**TABLE 2:** Spearman’s rank correlation analysis between SCUO and the three tAI measurements (original tAI, stAI, and gtAI) for the 12 model organisms.

		gtAI-SCUO (rho)	stAI-SCUO (rho)	tAI-SCUO (rho)
Archaea				
	*Ferroglobus placidus*	0.22*	0.24*	-
	*Halomicrobium mukohataei*	0.45*	0.27*	-
	*Methanocaldococcus jannaschii*	0.2*	−0.03	-
Bacteria				
	*Escherichia coli*	0.26*	0.27*	-
	*Neisseria meningitidis*	0.4*	0.22*	-
	*Vibrio cholera*	0.27*	0.28*	-
Eukarya (non-fungal)				
	*Caenorhabditis elegans*	0.26*	0.23*	-
	*Drosophila melanogaster*	0.6*	0.5*	-
Eukarya (fungal)				
	*Aspergillus fumigatus*	0.59*	0.49*	0.52*
	*Aspergillus nidulans*	0.48*	0.25*	0.45*
	*Saccharomyces cerevisiae*	0.41*	0.27*	0.36*
	*Schizosaccharomyces pombe*	0.22*	0.22*	0.20*

* represents *p* value <0.001.

### 3.4 The gtAI correlates better with PA data than stAI and CAI in both fungal and non-fungal organisms

The Williams’ test was used to compare the rho values at an alpha score of 0.01 (two-sided test). For *C. elegans*, *D. melanogaster*, *S. pombe*, *S. cerevisiae*, and *E. coli*, the gtAI showed a higher statistically significant correlation with PA than the stAI and CAI (Williams’ test *p* value <0.01). Furthermore, the gtAI exhibits a higher statistically significant correlation with PA in *E. coli* than the original tAI (Williams’ test *p* value <0.01). On the other hand, the original tAI predicted the PA of fungal organisms better than the gtAI and stAI, which is expected as it used experimental microarray data from yeast to obtain an optimal set of *S*_*ij*_ values that maximizes the correlation between expression levels and tAI values (dos Reis et al., 2004). Consequently, the gtAI is a valuable tool as it improves the prediction of PA in many organisms (Table 3).

**TABLE 3:** Spearman’s rank correlation analysis between PA and the three tAI measurements.

	gtAI-PA (rho)	stAI-PA (rho)	tAI-PA (rho)	CAI-PA (rho)
*Caenorhabditis elegans*	0.38*	0.36*	-	0.28*
*Drosophila melanogaster*	0.48*	0.44*	-	0.33*
*Escherichia coli*	0.54*	0.53*	0.5*	0.52*
*Saccharomyces cerevisiae*	0.50*	0.49*	0.56*	0.49*
*Schizosaccharomyces pombe*	0.61*	0.54*	0.62*	0.53*

* represents *p* value <0.001.

### 3.5 The absolute adaptiveness values generated by the gtAI reflect the evolutionary proximity

**FIGURE 2:**
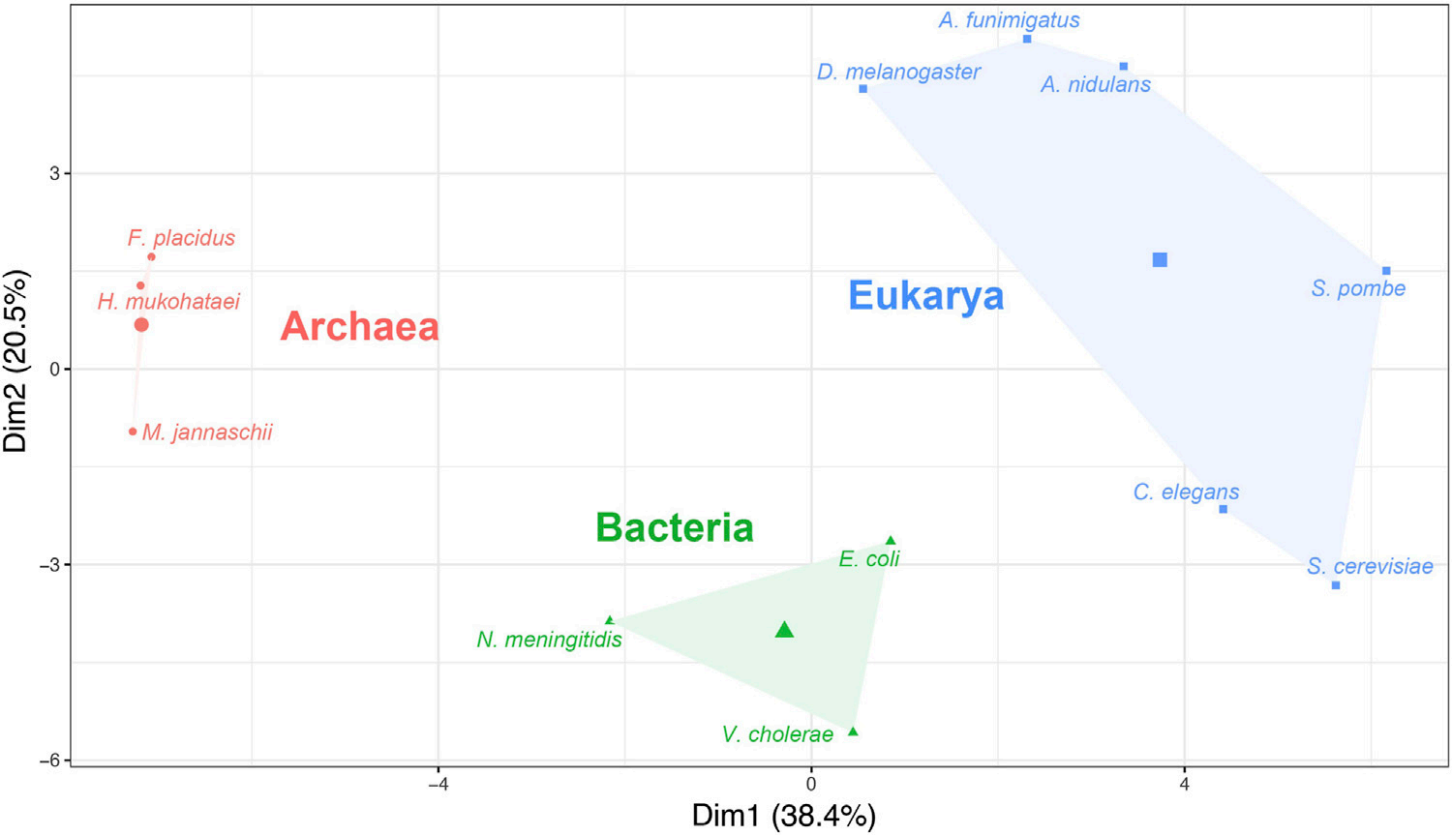
Hierarchical clustering on principal component (HCPC) analysis based on the absolute adaptiveness values (*W*_*i*_) of the 12 model organisms. The *x*-axis and *y*-axis represent the first and second principal components (Dim1 and Dim2), respectively (the clustering was performed using Ward’s method).

The absolute adaptiveness (*W*_*i*_) values of a codon depend on both the efficacy of codon–anticodon interaction (*S*_*ij*_ values) and the abundance of tRNA available for that codon. The number of tRNA genes and their abundance are diverse among the three domains of life (Fujishima and Kanai, 2014). Therefore, in theory, *W*_*i*_ should explain the divergence of organisms from different domains. To examine whether the *W*_*i*_ calculated using *S*_*ij*_ values generated by the gtAI are biologically meaningful, a hierarchical clustering on principal component (HCPC) analysis of *W*_*i*_ values was performed. The clustering classified all 12 model organisms used in the study into three distinct groups (Figure 2).

### 3.6 The effect of population size on gtAI result reproducibility

The inter-variability resulting from changing the population size in the three organisms was extremely low. For *S. cerevisiae*, *E. coli*, and *H. mukohataei*, the average best solution in the five experiments (the optimization of the non-parametric Spearman correlation between RSCU and *W*_*i*_ values) ranged from 0.8109 to 0.8134 (SD = from 0.0016 to 0.0031), from 0.6017 to 0.6022 (SD = from 0.001 to 0.0023), and from 0.4287 to 0.4308 (SD = from 0 to 0.0067), respectively. Then, the coefficient of variation (CV) for the same population size (10, 20, 30, n + 10, … , 100) was computed from the results of the five performed experiments for each organism. *S. cerevisiae* showed CV ranging from 0.00057 to 0.0042, *E. coli* ranging from 0.0012 to 0.0036, and *H. mukohataei* ranging from 0 to 0.0223. The coefficient of variation (CV) shows the extent of variability to the population’s mean. Therefore, as the variability decreases, the CV approaches zero. The CV values for the tested genomes showed an extremely low intra-variability, approximately equal to zero CV. Therefore, the inter-variability and intra-variability resulting from choosing different population sizes in the gtAI algorithm will not influence the reproducibility of the results. However, we recommend choosing a constant population size for the whole analysis.

### 3.7 The effect of generation time on gtAI result reproducibility

**FIGURE 3:**
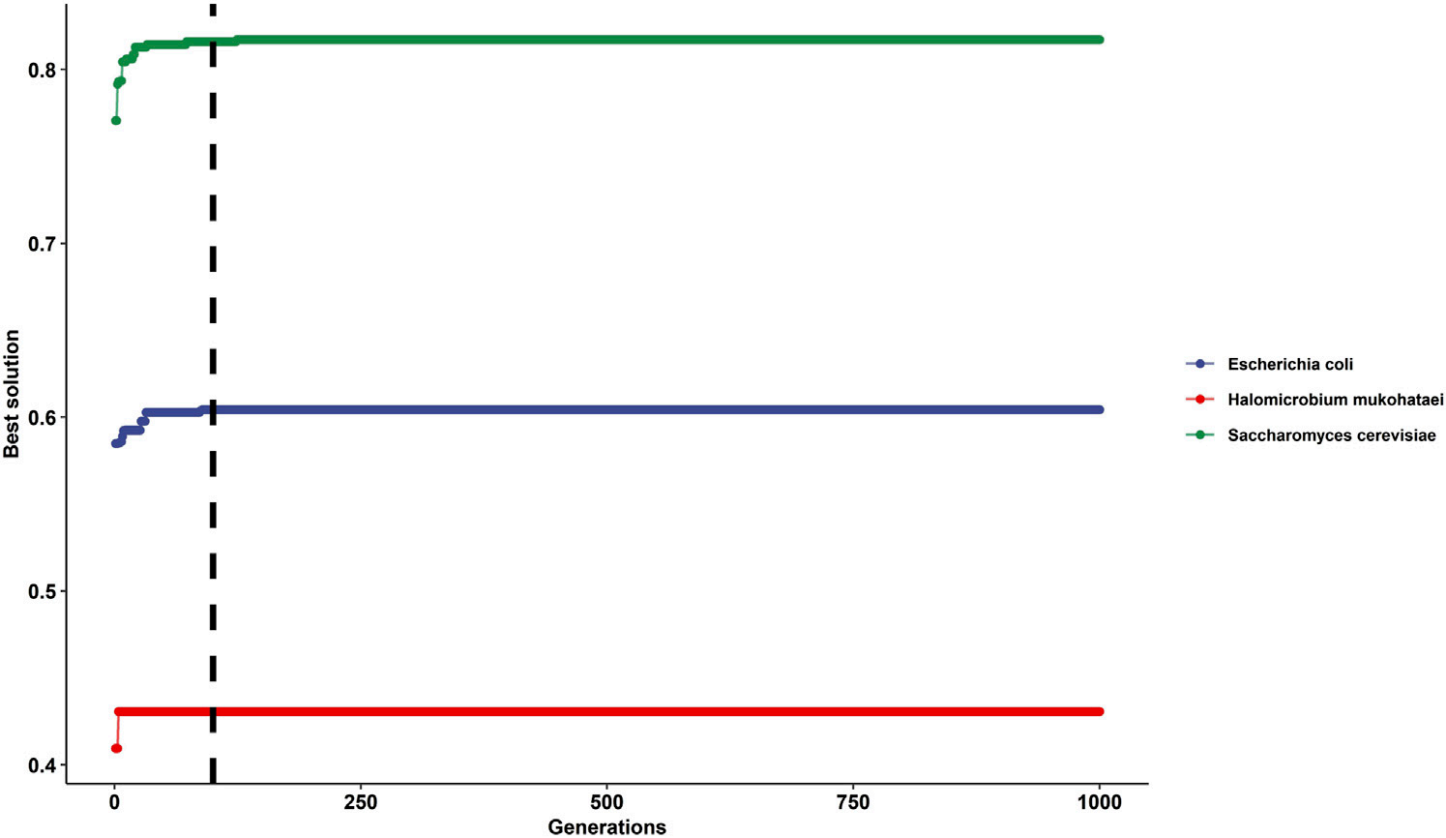
Impact of selecting different generation times for the genetic algorithm on gtAI results. The figure shows the best solutions after applying 1,000 generations in *Saccharomyces cerevisiae* (green line), *Escherichia coli* (blue line), and *Halomicrobium mukohataei* (red line). The *y*-axis represents the best solutions for optimizing the non-parametric (Spearman) correlation between RSCU (of the reference set) and *W*_*i*_ values at each generation. The *x*-axis represents the generation number—the vertical black line represents the generation time number 100 (the default generation time in the gtAI package).

At generation time 100 and higher, the solution was constant or had shallow changes (Figure 3). Therefore, we recommend using a generation time of 100 or higher, but a constant generation time must be selected for the whole analysis.

## 4 Discussion

The tAI is a formal measure of the force of translational selection. It has been widely employed to investigate fundamental questions related to gene expression, molecular evolution, and virus–host adaptation (Sharp et al., 2005; Man and Pilpel, 2007; Goodman et al., 2013; Pechmann and Frydman, 2013; Sabi and Tuller, 2014). Due to its importance, we were motivated to improve its performance by solving the issues of previous methods used for its calculation. We evaluated our proposed method mainly by examining whether it correlates better with other well-established codon usage indices. In addition, it shows a better association with empirical PA data.

The CAI is a gene-specific CUB index (Sharp and Li, 1987). Many studies suggested that the CAI is a good predictor of gene expression at mRNA and protein levels and has been used in many studies as a reference index to compare new indices and methods (Carbone et al., 2003; Sun et al., 2013; Fu et al., 2020). Accordingly, we conducted a comparative analysis on 12 model organisms (Supplementary Table S1) to evaluate the performance of the gtAI method compared to the original tAI and stAI (Supplementary Tables S2–S5) by examining whether it correlates better with the CAI using Spearman’s rank correlation analysis. The gtAI managed to outperform both methods by exhibiting a stronger significant correlation in 9 out of 12 model organisms with the CAI.

Attempting to explain the reason behind the better association of the stAI with the CAI than the gtAI in *F. placidus*, *V. cholera*, and *C. elegans* revealed a notable conclusion. These three organisms showed the highest ENc (low CUB) average value within their domains. For example, the average ENc value of the reference set for *F. placidus* was 44.8, while in *H. mukohataei* and *M. jannaschii*, the ENc values were 34.25 and 37.91, respectively. The same trend was observed in the bacterial group, as the average ENc value for *V. cholera* was 41.83, 39.08 for *E. coli*, and 37.08 for *N. meningitidis*. For non-fungal eukaryotes, *C. elegans* exhibited a 42.02 average ENc value, while 38.69 for *D. melanogaster*. This shows one limitation in our approach which can be attributed to organisms with overall weak CUB. It slightly influenced the result of the gtAI leading to the better correlation of stAI in these organisms. Furthermore, insights into the relation between GC content and gtAI performance have revealed that the change in GC content could not explain the slight outperformance of the stAI over the gtAI in terms of correlation with the CAI except in archaeal genomes. To embark on, in non-fungal eukaryotes, though *C. elegans* has an average GC content of 35.4%, indicating a possible strong bias against GC-rich codons, it showed an overall relatively weak bias (ENc = 42.02) which resulted in the slight underperformance of gtAI compared to stAI. Additionally, though *D. melanogaster* has a relatively higher GC content of 42.0% (closer to 50%), indicating relatively weaker bias, it showed an overall relatively stronger bias (ENc = 38.69) than *C. elegans*. Meanwhile, in Archaea, it is notable that the change in their overall bias is consistent with their deviation from the 50% GC content. In other words, the archaeal genome that showed an underperformance of gtAI (*F. placidus*) has an average GC content of 44.1% which is the closest to 50% compared to the other two archaeal genomes of 31.4% and 65.5%, as well as the highest ENc value of 44.8 compared to the other two of 34.25 and 37.91. Without regard to this limitation, the results (Table 1) suggest that the gtAI method performance was better for *S*_*ij*_ value optimization, giving a more reliable tAI value that better demonstrates the effect of translational selection.

One can argue that the correlation between the gtAI and CAI might be inflated due to using the same reference set of genes. Hence, we conducted two more analyses to test it. In the first one, we obtained multiple random samples of CAI and the three tAI indices’ values and performed Spearman’s rank correlation analysis between each of them with the CAI. The results remained the same and agreed with our conclusion as the gtAI showed a stronger correlation with the CAI than the stAI in the same nine organisms. In addition, a higher correlation with the CAI than the original tAI in the four fungal organisms was shown. In the second analysis, we investigated whether gtAI correlates better with another CUB index that is independent of using a reference set of genes in its calculation, namely, SCUO (to exclude the reference set parameter). The results also revealed a stronger gtAI association with SCUO, further confirming that the correlation between the gtAI and CAI is not inflated nor affected by the reference set of genes.

In conclusion, our gtAI method can solve the query of *S*_*ij*_ value optimization and effectively estimate the tAI values while overcoming the limitations observed in other implementations. Performance evaluation showed that the gtAI method performed better than the original tAI and stAI by exhibiting a stronger correlation with the CAI and SCUO. It has also improved the prediction of PA compared to the stAI and CAI. The reproducibility of the genetic algorithm employed by the gtAI was tested and revealed its reliability in reaching the best solution in complex optimization problems. The *Wi* values generated by the gtAI correctly reflect the evolutionary proximity between organisms from different domains of life. Indeed, one significant advantage of CUB-dependent tAI computation methods (i.e., gtAI and stAI) over the original tAI is the lack of neediness for external information such as gene expression data or mRNA levels (which are often unavailable for most genomes). We believe that the gtAI will allow for obtaining higher quality tAI results used to draw conclusions about the force of translational selection acting on genes in related studies.

## Data Availability

The original contributions presented in the study are included in the article/Supplementary Material; further inquiries can be directed to the corresponding author.
