# Supplementary figures and images for: gtAI: an improved species-specific tRNA adaptation index using the genetic algorithm
Source: Front Mol Biosci. 2023 Jul 4;10:1218518. doi: 10.3389/fmolb.2023.1218518 (PMC10352787; doi:10.3389/fmolb.2023.1218518)

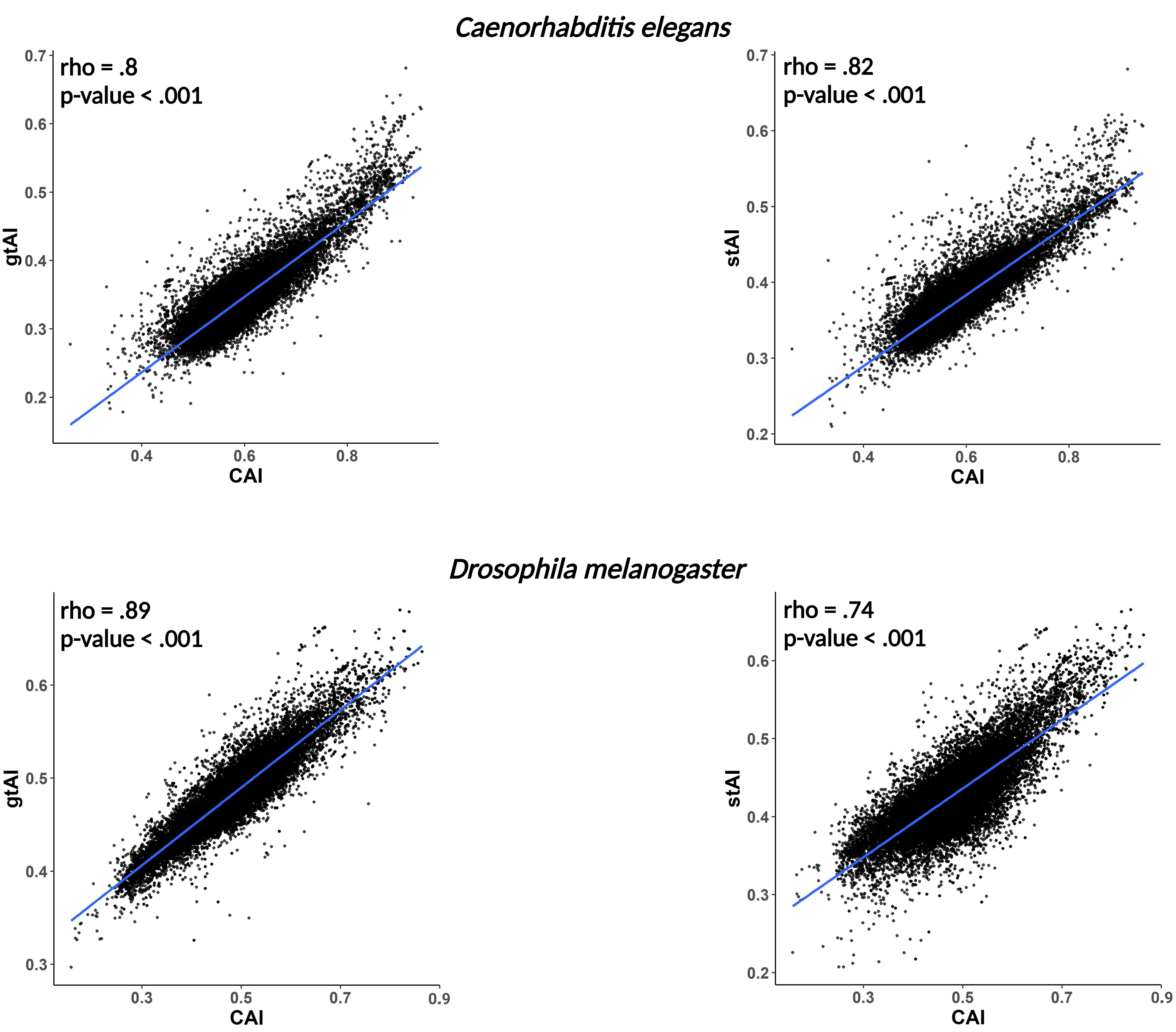

Supplement: Supplementary file 3 [file Image4.PNG]

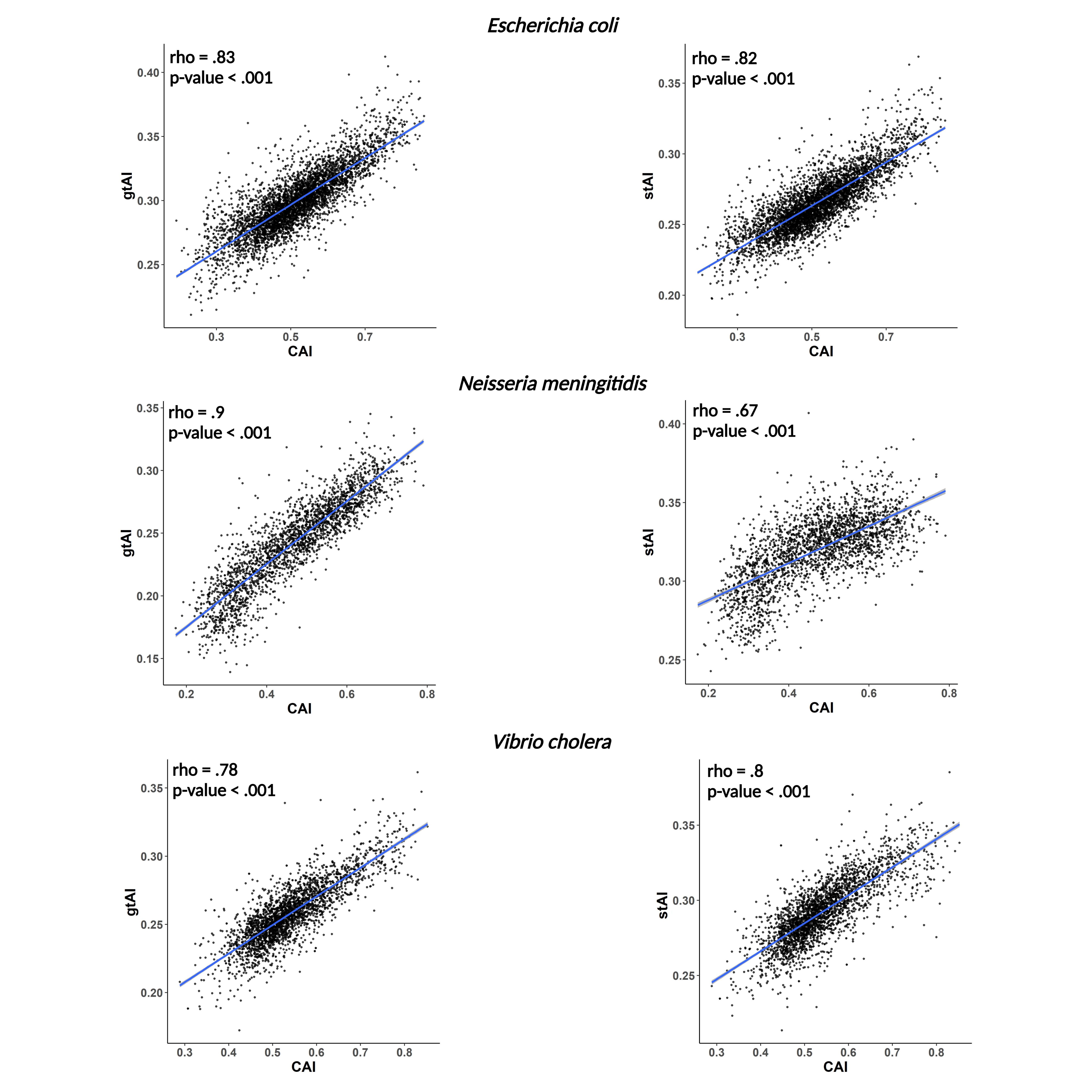

Supplement: Supplementary file 5 [file Image2.PNG]

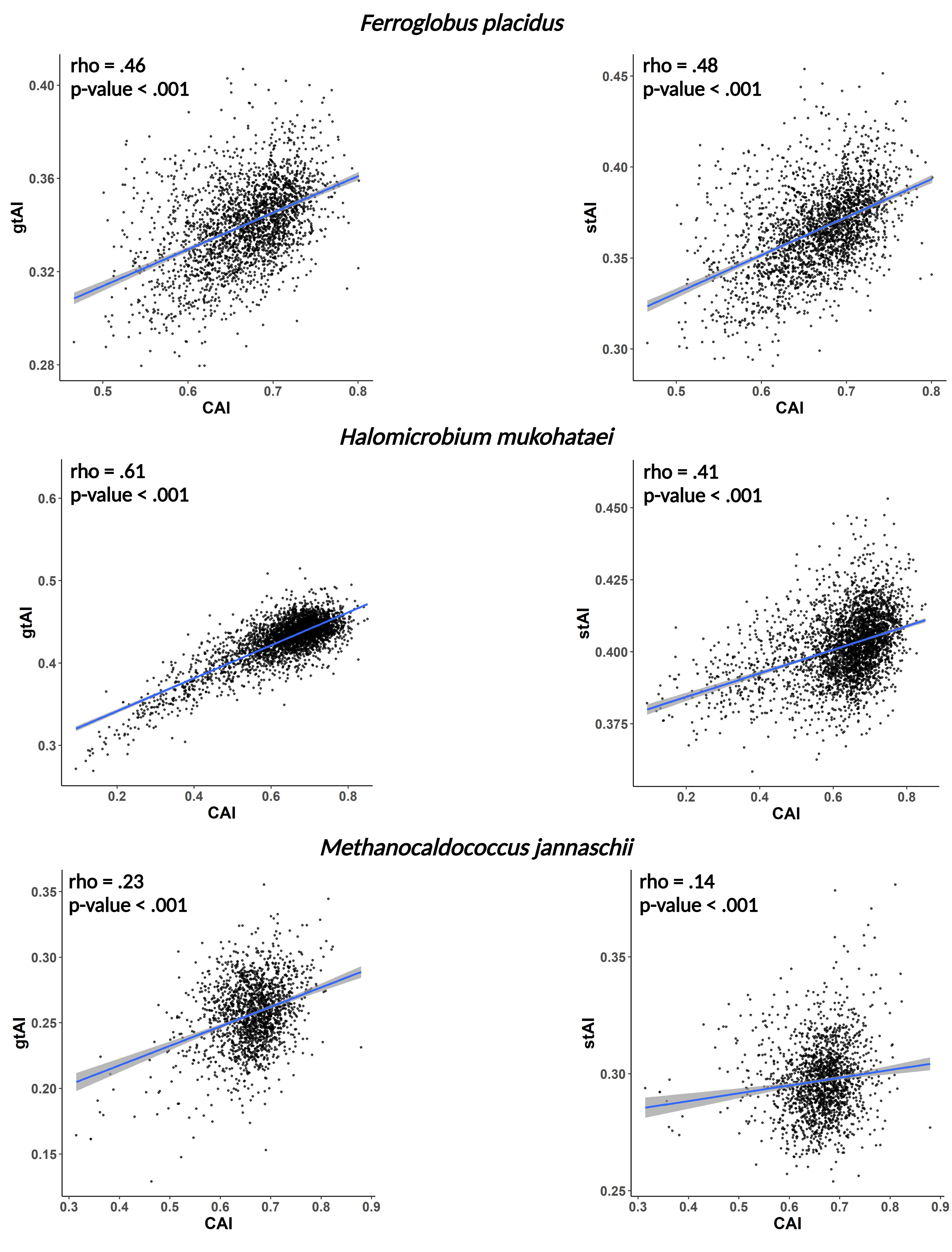

Supplement: Supplementary file 7 [file Image1.PNG]

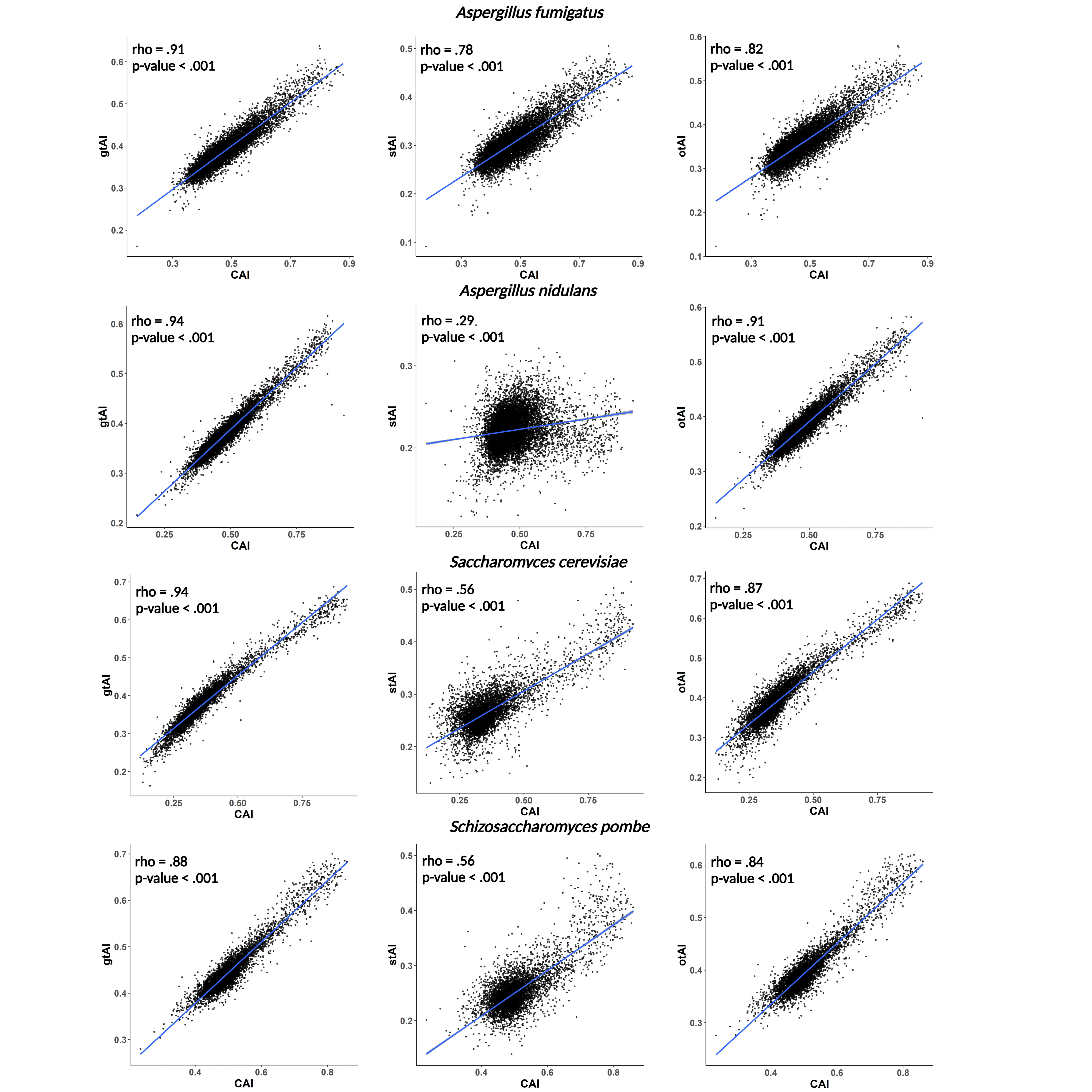

Supplement: Supplementary file 9 [file Image3.PNG]
